# A Comparative Study for Material Selection in 3D Printing of Scoliosis Back Brace

**DOI:** 10.3390/ma15165724

**Published:** 2022-08-19

**Authors:** Alfredo Ronca, Valentina Abbate, Davide Felice Redaelli, Fabio Alexander Storm, Giacomo Cesaro, Cristina De Capitani, Andrea Sorrentino, Giorgio Colombo, Paolo Fraschini, Luigi Ambrosio

**Affiliations:** 1 Institute of Polymers, Composites and Biomaterials (IPCB), National Research Council of Italy, Via Previati 1/C, 23900 Lecco, Italy; 2 Department of Chemical, Materials and Industrial Production Engineering (DICMaPI), University of Naples Federico II, Piazzale V. Tecchio 80, 80125 Napoli, Italy; 3 Institute of Intelligent Industrial Systems and Technologies for Advanced Manufacturing (STIIMA) National Research Council of Italy, Via Previati 1/C, 23900 Lecco, Italy; 4 Scientific Institute IRCCS E. Medea, Via Don Luigi Monza 20, 23842 Bosisio Parini, Italy; 5 Institute of Polymers, Composites and Biomaterials (IPCB), National Research Council of Italy, Via Campi Flegrei, 34, 80078 Pozzuoli, Italy; 6 Department of Mechanical Engineering, Politecnico di Milano, Via G. La Masa, 1, 20156 Milano, Italy

**Keywords:** additive manufacturing, functional characterisations, advanced material selection, orthopaedic back braces

## Abstract

In recent years, many research studies have focused on the application of 3D printing in the production of orthopaedic back braces. Several advantages, such as the ability to customise complex shapes, improved therapeutic effect and reduced production costs place this technology at the forefront in the ongoing evolution of the orthopaedic sector. In this work, four different materials, two of them poly(lactic acid) (PLA) and two of them poly(ethylene terephthalate glycol) (PETG), were characterised from a thermal, mechanical, rheological and morphological point of view. Our aim was to understand the effects of the material properties on the quality and functionality of a 3D-printed device. The specimens were cut from 3D-printed hemi-cylinders in two different orientation angles. Our results show that PETG-based samples have the best mechanical properties in terms of elastic modulus and elongation at break. The PLA-based samples demonstrated typical brittle behaviour, with elongation at break one order of magnitude lower. Impact tests demonstrated that the PETG-based samples had better properties in terms of energy absorption. Moreover, 3D-printed PETG samples demonstrated a better surface finishing with a more homogenous fibre–fibre interface. In summary, we demonstrate that the right choice of material and printing conditions are fundamental to satisfy the quality and functionality required for a scoliosis back brace.

## 1. Introduction

**Figure 1:**
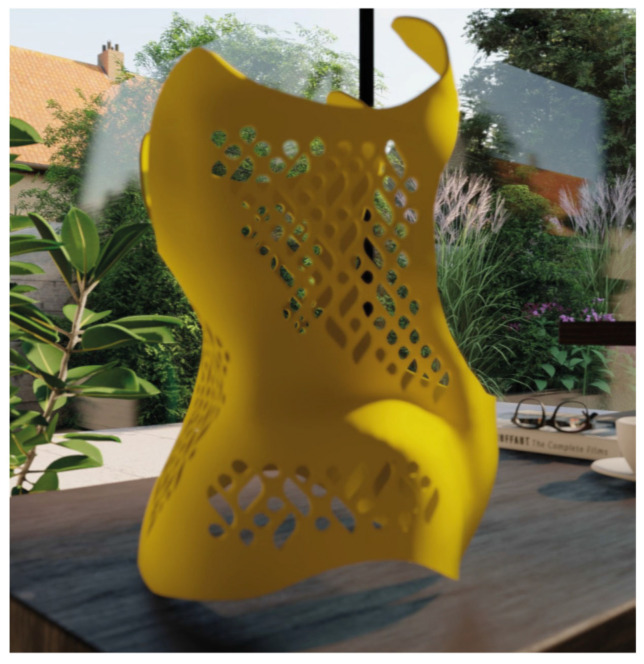
An example of a textured 3D-printed brace realised at the IPCB-Lecco lab (EMPATIA@LECCO, http://www.ipcb.cnr.it, accessed on 20 June 2022).

Millions of people around the world suffer from a condition known as idiopathic scoliosis (IS), which is the rotational or lateral curvature of the spine. A back brace is effective for patients with IS and its efficacy is significantly correlated with the average daily time of wearing [1,2]. Various methods of producing orthopaedic back braces are currently used [3,4]. The majority of these techniques are based on manual thermoforming of thermoplastic sheets [5]. Several advantages, such as the simplicity of production and the cost-effectiveness of equipment and raw materials, make this manufacturing process the preferred approach by the operators of this sector. However, manual operations do not allow for precise control over the thickness of the back braces, nor do they allow for textures and shapes capable of meeting a good level of aesthetics, functionality and comfort [6,7]. In recent years, the introduction of computer-aided design and novel manufacturing methods has significantly increased the quality and speed of brace making [8,9,10]. Among these systems, additive manufacturing can potentially allow for further improvements to the functionality and aesthetics of these medical devices (Figure 1), which also increases patient acceptance and therapeutic effects [11,12,13]. However, current knowledge regarding the design and manufacture of a 3D-printed brace adapted to the type of scoliosis and the patient’s personal needs is still scarce [14,15,16]. This is particularly true when it comes to the choice of material and the optimal processing conditions for making a back brace [17,18].

The principle underlying all additive technologies is the same: a virtual model of the object to be realised is divided into a series of discrete 2D layers, which in turn become a series of sequential commands that a printer uses to precisely deposit a material, layer by layer, on a building plate [19,20,21]. The virtual model can be quickly obtained, with the desired level of precision, from a 3D scan of the patient’s body and used as a template for designing the correct device configuration [21,22,23]. Modern 3D printing technology allows for the use of materials in any form (solid, powder, melt and liquid) to obtain different levels of resistance and precision [15,20]. Among these technologies, fused deposition modelling (FDM™) is probably the most interesting for its reliability, cost-effectiveness and ease of use [24]. In particular, when the objects to be made have medium/large dimensions, the FDM™ process provides a good compromise between final resolution, printing speed and material cost. Furthermore, this process has the advantage of being able to treat numerous polymeric and composite materials with a wide range of properties [19,25]. In its traditional configuration, thermoplastic material in the form of a filament with a constant diameter is melted and pushed through an extrusion nozzle. The size of the nozzle determines the thickness of the output layers, the printing time, and the final resolution of the object [26]. Despite offering several advantages, the application of FDM™ technology has generally been limited to prototypes [24,27,28]. Up until now, the anisotropic properties of the printed parts have severely limited their application in the production of functional objects [29,30,31]. In the growth direction, where the continuity of the part is guaranteed only by the adhesion between the deposited layers, mechanical properties are strongly dependent on the processing conditions, such as extrusion temperature, layer thickness and printing speed [32,33,34,35]. Many studies in the literature have focused on the optimisation of the process parameters and their relationship with the final properties of the 3D-printed parts [31,36].

Another important limitation to the use of 3D printing for practical applications is related to the availability of material suitable for the specific applications [25,37]. Regarding the FDM™ process, poly(lactic acid) and poly(ethylene terephthalate glycol) are the most popular, cheap and simple-to-use thermoplastic materials currently available for FDM™. Poly(acrylonitrile-butadiene-styrene) (ABS), polyamide (PA), polycarbonate (PC) and thermoplastic polyurethane (TPU) are other polymeric matrices available on the market. However, these materials present several limitations such as high extrusion temperature (PA and PC), low mechanical modulus (TPU), toxic chemicals produced during the melting process (ABS, PC), high density (PA and PC), high level of shrinkage (PA and ABS), high content of unsafe fillers and additives (PA and PC) and high cost (PA, PC, TPU).

Despite the number of filaments available on the market, information on their properties is often limited, which makes it difficult to compare different data sheets. Filament manufacturers often make use of different test standards and/or provide incomplete characterisations. Furthermore, the data provided often refers to the raw material (Table 1), while the mechanical characterisation of the printed samples is very poor. Variabilities within molecular weight and distribution, viscosity, crystallinity and additives are not reported. Accurate property measurements are necessary to make material decisions in safety-critical designs such as biomedical applications. Additional research works are necessary to develop test standards based on the final usage of the material, inherent weaknesses in the design, durability requirements and safety factors [38].

To select the appropriate material for a specific application, it is essential to take into account both the amount of mechanical and environmental stresses that the application will require and the geometric constraints in which the object must be printed. This is particularly true for orthopaedic applications. Specialised properties such as toughness, wear resistance, moisture absorption and biocompatibility are essential requirements for orthopaedics and surgical devices. The choice of material is a compromise between the available materials and the limits of the printing technology in use. Parameters such as mechanical strength, temperature of use, degree of finish and cost must be carefully evaluated to ensure the desired result. Without doubt, the choice of material is the most delicate phase when developing a new medical device.

The lack of standards for FDM™ manufacturing and testing has led to incongruent conclusions of test results and print settings. Many studies in the literature have focused on the optimisation of the process parameters and their relationship with the final properties of the 3D-printed parts [36,39,40,41,42,43,44]. Recently, Morandi et al. conducted a systematic experiment to investigate the influence of infill-patterns (IPs) on specific mechanical responses of PLA parts fabricated using FDM™ [40]. Similarly, Rankouhi et al. attempted to address the debatable effects of layer thickness on the mechanical properties of 3D-printed ABS samples using FDM through a set of extensive tensile tests followed by statistical analysis of the results [41]. Rodriguez et al. utilised an integrated process–materials–design methodology to optimise the mechanical properties of parts fabricated using FDM™ for raster orientation aimed at moving FDM™ into volume production of functional component domains [42]. Patti et al. focused on the characterisation of 3D-printed parts made from a composite filament, highly loaded with stainless steel microparticles and prepared at different infill densities (0, 50, 100%) [43]. Urquiza et al. prepared new green composites through 3D printing PLA onto jute fabrics to evaluate mechanical performance, allowing for the discovery of multiple industrial applications [44]. Lee et al. attempted to describe the optimisation of FDM™ process parameters for the optimum performance of compliant ABS prototypes in terms of elasticity and flexibility to realise a model of a catapult that could be used in a sling shot toy [36].

In this work, a different approach was used to perform a complete characterisation in terms of the mechanical, morphological, rheological and thermal characteristics of 3D-printed samples realised with two different commercial polymers (PETG and PLA). For the characterisation process, the samples were not directly 3D printed but instead cut from a hemi-cylinder that, due to its particular shape, was representative of a scoliosis back brace. In this way, it was possible to have an overview of the correlation between printing parameters and the final properties of the back brace. The goal was to carry out a realistic study on the correlation between processing conditions such as layer and wall thickness, filament deposition angle and material properties with the final properties of the 3D-printed parts.

## 2. Materials and Methods

### 2.1. Materials

Two different polymer matrices (PLA and PETG) and two commercial brands for each polymer were considered: filaments from TreeD Filaments^®^ and Filoalfa^®^ for PLA, and filaments from Sunlu^®^ and Filoalfa^®^ for PETG. The following Table 1 summarises all properties provided by the suppliers in the respective datasheets/websites.

### 2.2. Samples Preparation

**Figure 2:**
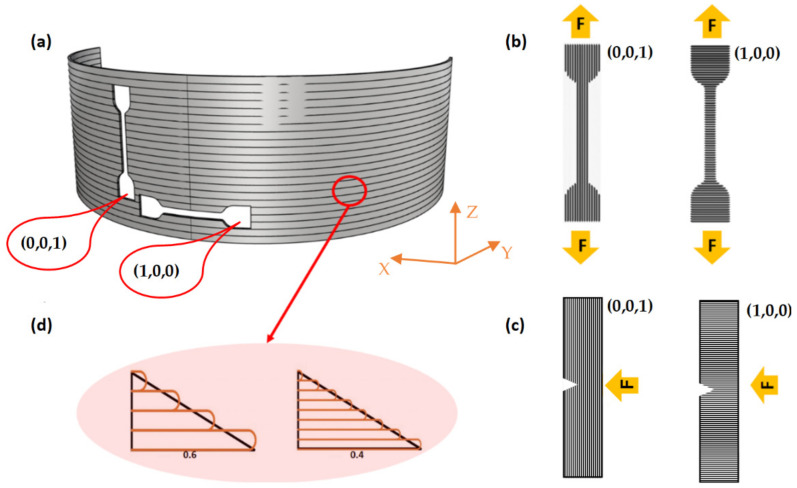
(**a**) Schematic representation of the test specimens cut from hemi-cylinder; filament orientation for (**b**) tensile and (**c**) Charpy impact test; (**d**) layer thickness considerations for the realisation of the hemi-cylinder.

A hemi-cylinder geometry similar to the section of the dorsal brace was chosen for the preparation of the samples (Figure 2a). The dimensions were 150 mm in diameter, 50 mm in height, and 2 mm in thickness. The main advantage of this geometry concerns the possibility of testing the properties of the structure in its final configuration, additionally considering the effect of the real process conditions and the different directions with respect to the deposition of the layers [17]. The sample model was created with the CAD software Rhinoceros and exported as an STL file. The STL file was then imported into Ultimaker Cura, which performed both the slicing and the gcode file creation. The samples were printed using an FDM™ delta type machine (WASP 4070, WASP, Massa Lombarda, Italy) with a 1.0 mm circular nozzle. The following printing parameters remained constant during all experiments: 60 °C heated bed temperature, 240 °C extrusion temperature and 100 mm/s printing speed. Two layer heights (0.4 and 0.6 mm) were selected as input factors. During the process, the printer proceeded by describing an arc while depositing layer by layer of two closely spaced 0.6-mm-thick fused filaments, one going forward and the other returning. The samples were cut in the central position of the hemi-cylinder, where the deposition time between one filament and the next was maximum and constant throughout the printing process. The hemi-cylinder was oriented on the building plate in such a way as to be optimally exposed to the flow of air produced by the cooling fans.

### 2.3. Rheological Analysis

A stress-controlled rotational rheometer (HAAKE RheoScope MARS III, Thermo Fisher Scientific, Waltham, MA, USA) equipped with 20 mm parallel plates was used for the rheological characterisation of the samples. Shear tests were carried at 220 °C under a dry nitrogen atmosphere. Specimens for the rheological characterisations were cut from the printed hemi-cylinder samples and vacuum dried at 60 °C for 8 h. In order to characterise the linear viscoelastic response of the materials, preliminary strain sweep tests were carried out at a constant frequency of 1 Hz. Frequency sweep tests (0.01–100 Hz) at a fixed strain of γ = 0.1 were performed to obtain the linear viscoelastic data.

### 2.4. Differential Scanning Calorimetry

The thermal properties were evaluated using differential scanning calorimetry (DSC—TA Instrument Q2000). The specimens (7–12 mg in weight) were subjected to a multiple DSC scan test consisting of a first heating from 30 °C to 200 °C at 10 °C/min followed by a cooling from 200 °C to −20 °C at 10 °C/min and finally a heating up to 200 °C at 10 °C/min.

### 2.5. Tensile Test

The tensile properties were evaluated at room temperature using a dynamometer equipped with a load cell of 1 kN. As depicted in Figure 2, dumbbell-shaped specimens (according to the ASTM D1708 standard) were cut from the 3D-printed hemi-cylinder in two different orientations: parallel (1,0,0) and orthogonal (0,0,1) to the layer deposition. The test specimens reported in Figure 2b were subjected to tensile strength testing until rupture at a crosshead speed of 1 mm/min. Two different layer thicknesses (namely 0.4 and 0.6 mm) were considered (Figure 2d). The elastic modulus (E′) value was determined by considering the first linear portion of the stress–strain curve (between 0.05% and 0.20% strain) and estimating its slope using linear fitting procedures. Following the ASTM D1708 standard, the sample strength (*σ_y_*) was computed as a ratio between the yield load in the test and the full cross-sectional area of the specimen following Equation (1) [45]:

(1) σy=Fyb·h

Both *σ_y_* and E′ values were estimated as averages over five samples.

### 2.6. Charpy Impact Test

Charpy impact tests were performed to evaluate the shock absorption capacity of the samples obtained with the different materials. The impact tests were carried out at room temperature with a Charpy Ceast Resil impactor equipped with a CEAST Mod. 6545 acquisition system (impact energy = 3.6 J, speed = 1 m/s). For each analysed condition, 5 samples measuring 10.0 × 3.5 × 60 mm^3^ were tested. All samples had a notch depth-to-width ratio of 0.3 and a span length of 48.0 mm. The energy absorbed by the samples during fracture was determined by Equation (2) [46]:

(2) $$E_{T}=m\cdot g\cdot \left(h_{0}-h_{f}\right)$$

in which *m* is the mass of the pendulum and *h*_0_ and *h_f_* are the starting and final heights of the pendulum, respectively. Figure 2c reports the experimental results for the two different sample orientations: parallel (1,0,0) and orthogonal (0,0,1) to the impact force. The absorbed energy per unit cross-sectional area (kJ/m^2^) or impact strength (*Ec*) was determined by Equation (3) [47]:

(3) Ec=ETw·t

where *w* and *t* are the width and the thickness of the specimen, respectively.

### 2.7. Dynamic Mechanical Thermal Analysis (DMTA)

Mechanical properties at different temperatures were determined by means of a dynamic mechanical analyser (DMA 2980, TA Instruments, New Castle, DE, USA). Rectangular samples (10 × 35 mm) were cut in the orthogonal (0,0,1) direction from the hemi-cylinder (Figure 2). Samples were tested by applying a variable flexural deformation at a frequency of 1 Hz. The displacement amplitude was set to 0.1% while the temperature was increased from 25 to 160 °C at the scanning rate of 3 °C/min.

### 2.8. Density Measurements

Apparent density measurements were made using a microbalance for weight measurements and digital callipers and a micrometre for dimensional measurements. The bulk density was measured with a hydrostatic balance according to ASTM D792. The macro-porosity ∅ of each sample was determined using Equation (4) [48]:

(4) $$\emptyset =\left(1-\frac{\delta g}{\delta b}\right)$$

where *δg* is the apparent density and *δb* the bulk density. The experimental results were obtained as the average of three specimens for each analysed condition.

### 2.9. Scanning Electron Microscopy (SEM)

The sample surface analysis of the samples was carried out with a scanning electron microscope (SEM) (FEI Quanta 200 FEG, Thermo Fisher Scientific, Waltham, MA, USA) operating at 20 kV. The samples were coated with gold with a sputter coater (SC500, Emscope, Hertfordshire, UK).

## 3. Results

### 3.1. Rheological Analysis

**Figure 3:**
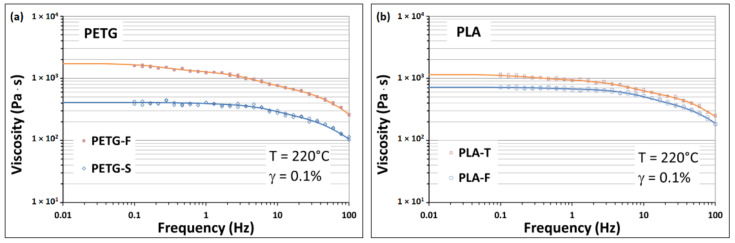
Rheological characterisation of (**a**) PETG and (**b**) PLA samples.

The 3D printability of a polymeric material refers to its ability to be extruded in the form of continuous filaments, hold the printed shape, form stable multilayer structures and eventually form bridges without sagging or collapsing. These characteristics are closely related to the material’s molecular structure and the presence of additives and fillers. The printability can be evaluated based on various rheological parameters including elasticity and viscosity behaviour [49]. In this work, the rheological behaviour of the materials was evaluated using dynamic frequency scanning tests; the experimental results are shown in Figure 3a,b. Complex viscosity as a function of frequency shows a fairly similar trend for all the materials analysed. In particular, all the curves show a Newtonian behaviour of the material at low-medium levels followed by a shear-thinning behaviour at the highest frequencies. The reduction of the complex viscosity at high frequencies is attributed to disentanglement of polymer chains when high deformation rate is applied to the melt. This type of behaviour is quite common for materials used in the FDM™ process, in which high fluidity is required in the initial stages of extrusion from the nozzle (at high deformation rates) followed by a rapid increase in viscosity with a simultaneous mechanical stabilisation of the piece (at low deformation rates) in the deposition/cooling phase. High material viscosity can cause the filament to buckle. This phenomenon occurs when the required extrusion pressure is greater than the critical load that the filament can support [50].

It has been shown that the ratio between the compressive elastic modulus of the material at room temperature and its viscosity at the extrusion conditions provides a good estimation of the degree of printability of the material. In other words, at constant elastic modulus, the lower the viscosity of the material at the considered temperature and extrusion speed, the lower the chances of an instability of the filament during printing.

From this point of view, it is clear from Figure 3 that PLA-F and PETG-S materials must be preferred over the other two as they have a greater stability range and therefore allow printing at lower temperatures and/or at faster speeds. However, heavy non-Newtonian characteristics promote a significant swelling of melt at the nozzle exit with possible shape distortions of the printed parts. High viscosity under zero shear conditions also guarantees the absence of dripping phenomena during a pause in extrusion and part stability during the deposition and cooling phase. In our case, the absence of a rise in viscosity at low frequencies is indicative of the fact that none of the materials investigated contain additives or inorganic fillers capable of modifying the relaxation dynamics of the chains [49].

### 3.2. Thermal Proprieties

**Figure 4:**
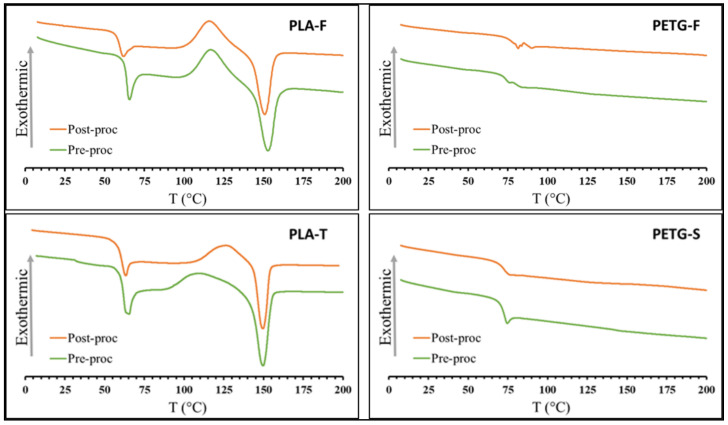
DSC analysis of PETG and PLA pre- and post-processed samples.

The effect of the process on the thermal properties of the 3D-printed samples was investigated by means of DSC tests. The melting and crystallisation behaviour of polymers is not only important for evaluating the sample morphology but also has a crucial influence on the mechanical stability of the printed parts. Amorphous and slowly crystallising polymers are generally preferred in 3D printing over fast-crystallising polymers such as polypropylene and polyethylene. In fact, fast solidification during the filament deposition does not allow sufficient welding within the layers and leads to a high level of shrinkage. The last is certainly a challenge since this could seriously warp the printed objects during cooling, and this reason considered, two amorphous (PETG) and two slow-crystallising polymers (PLA) were chosen for the 3D printing of braces. Figure 4 shows the DSC graphs of heat flux (W/g) versus temperature (°C) for the PLA and PETG filaments (pre-processing) and 3D-printed specimen (post-processing). Glass transition temperature (T_g_), melting temperature (T_m_) and enthalpy of fusion (ΔH_m_) are reported in Table 2. The thermogram of PLA shows the three typical peaks of semi-crystalline thermoplastics, from left to right: an endothermic peak associated with the enthalpic relaxation at T_g_, an exothermic peak associated with cold crystallisation, and an endothermic peak associated with the crystals melting. From the DSC curves of both the pre- and post-processed PLA materials, it is possible to assess that only small differences are observed. This result suggests that the physical properties of the materials did not change during the printing process. In other words, it means that the materials analysed undergo thermal degradation at temperature well above the extrusion temperature [51]. Some differences can be found in the melting behaviour of pre- and post-processing PLA. For example, the enthalpy of fusion of PLA-F was found to be ΔH_m_ = 23.95 J/g for the post-processed materials and ΔH_m_ = 14.95 J/g for the pre-processed material. This difference can be attributed to the formation of an additional crystalline phase that can form during the printing process [52]. As shown in Table 1, similar behaviour was found in the PLA-T samples.

In both PLA materials, the raw material and the printed samples show clear evidence of a cold crystallisation process at temperatures above the T_g_. For PLA-T samples, these exothermic peaks are noted to shift from 117 °C to about 120 °C after printing. In this case, the printing process seems to induce a slight crystallisation during cooling. In any case, the cold crystallisation peak evidences a non-complete crystallisation of the materials during the process. This also means that the printed PLA structures will not be stable over time as they will be subjected to a gradual crystallisation that could induce fractures and uncontrolled warpage [53,54].

The thermogram of PETG materials shows the typical behaviour of an amorphous polymer with only a T_g_ transition at about 70°C [55]. Additionally, in this case, the thermal behaviour pre- and post-processing are quite similar.

### 3.3. Mechanical Properties

#### 3.3.1. Tensile Test

**Figure 5:**
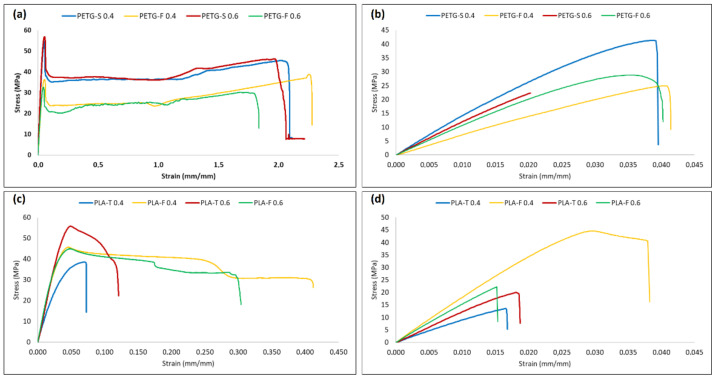
Stress–strain curves for PETG and PLA samples in (1,0,0) (**a**,**c**) and (0,0,1) (**b**,**d**) direction.

An extensive mechanical characterisation was conducted to study the effects of the material and the print orientation on the modulus and strength of the printed samples. Two different layer thicknesses were considered, 0.4 and 0.6 mm. Figure 5 reports the stress–strain curve for PLA and PETG samples in the (1,0,0) direction (Figure 5a,c) and (0,0,1) direction (Figure 5b,d). The results clearly show a strong mechanical anisotropy of the samples while layer thickness seems to have limited influence on the mechanical properties. From Figure 5 it is clear that 3D-printed PLA-based samples (Figure 5a,b) have inferior mechanical proprieties when compared to PETG samples (Figure 5c,d).

**Figure 6:**
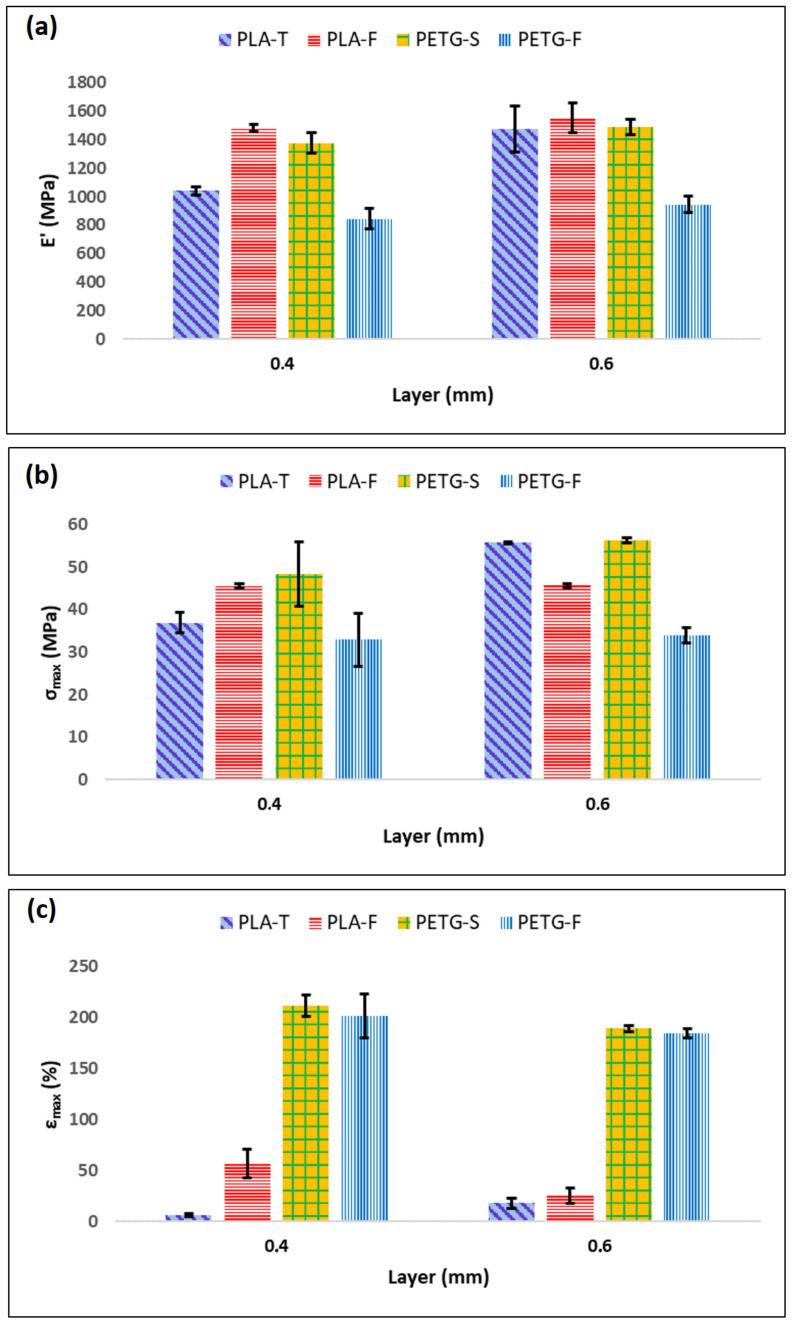
Mechanical properties of PLA and PETG samples in (1,0,0) direction as a function of the layer thickness. (**a**) represents the elastic modulus (E′), (**b**) the yield strength (σ_max_) and (**c**) the elongation at break (ε_max_).

**Figure 7:**
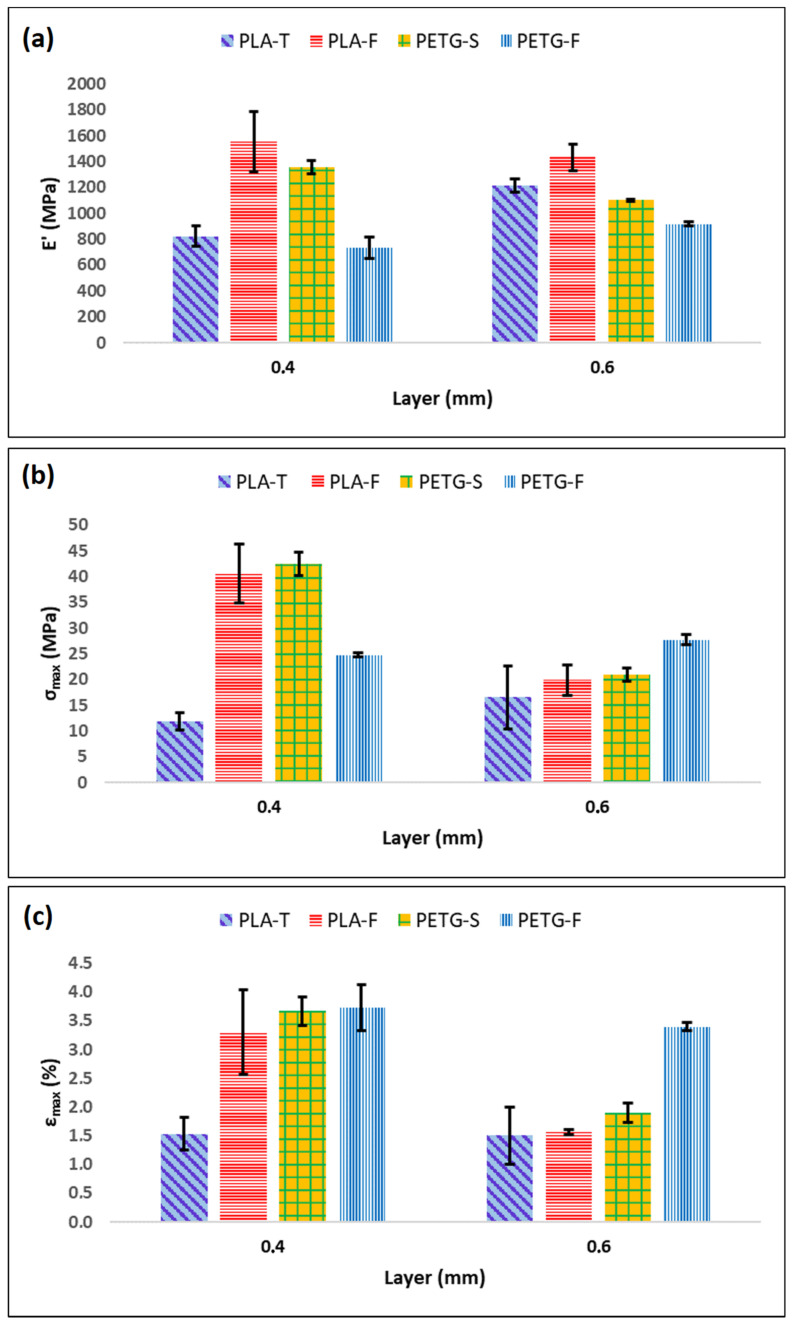
Mechanical properties of PLA and PETG samples in (0,0,1) direction as a function of the layer thickness. (**a**) represents the elastic modulus (E′), (**b**) the yield strength (σ_max_) and (**c**) the elongation at break (ε_max_).

To better understand the correlation between the different materials, the means and standard deviations of the test results (elastic modulus (E′), yield strength (σ_y_) and elongation at break (ε_max_)) are provided in Figure 6 and Figure 7. In particular, Figure 6 shows E′, σ_max_ and ε_max_ for PETG and PLA when the filament angle is aligned with the applied forces. Starting from Figure 6a, it is possible to assess that there are only minor differences between PETG-S and PLA-F that show an elastic modulus of 1377.30 ± 73.63 MPa and 1480.93 ± 23.85 Mpa, respectively. Conversely, PETG-F and PLA-T filaments show a lower elastic modulus of 899.66 ± 54.11 MPa and 1040.93 ± 28.70 MPa, respectively.

σ_max_ follows the same trend of E′, with higher values for PETG-S and PLA-F (48.30 ± 7.60 MPa and 45.58 ± 0.54 MPa respectively) (Figure 6b). However, the situation changed for ε_max_ (Figure 6c). In fact, when the applied force was parallel with the fibre direction, both PETG-S and PETG-F demonstrated an elongation at break one order of magnitude higher compared to PLA samples. PETG demonstrated a typical elasto-plastic behaviour with a first elastic region followed by a plastic deformation before break (Figure 5a). Comparatively, PLA demonstrated a brittle behaviour with a first elastic region followed by the sample breaking (Figure 5c). Looking at the effect of layer thickness on the mechanical properties in the (1,0,0) direction, it is possible to say that both the 0.4 and 0.6 mm layer have the same mechanical behaviour with comparable elastic modulus, yield stress and elongation at break, both for PETG and for PLA samples.

Again, when the force was applied orthogonal to the printing direction (Figure 5b,d) the samples show a quite different behaviour. In fact, it is possible to see from Figure 5b that PETG samples show a typical brittle behaviour with a fragile break after the elastic region.

The elastic modulus remains similar both for PETG and PLA samples with values of 1358.17 ± 54.21 MPa and 1557.50 ± 233.06 MPa for PETG-S and PLA-F 0.4 mm, respectively. Likewise, the yield stress shows similar behaviour in T direction with values of 42.44 ± 2.34 MPa and 40.58 ± 5.73 MPa for PETG-S and PLA-F 0.4 mm thick, respectively.

However, for PETG samples the elongation at break decreases significantly, from 211.0 ± 10.8% in the (1,0,0) direction to 3.6 ± 0.25% in the (0,0,1) direction. In addition, PLA samples have different behaviour in terms of elongation at break if the (0,0,1) and (1,0,0) direction are compared. In fact, for example, PLA-F in the (1,0,0) direction has the best elongation at break with a value of 56.3 ± 13.9% that becomes 3.3 ± 0.7% in the (0,0,1) direction, with a reduction of one order of magnitude.

This difference between filament orientations can be explained by considering inter-layer adhesion bonds and the tensile strength of each individual bond, known as trans-layer strength. The inter-layer adhesion failure has the least influence on mechanical strength of specimens in the (1,0,0) direction, since each fibre is pulled along its longitudinal axis. For the samples in the (0,0,1) direction, however, the force was applied perpendicular to the direction of the fibres. In this case, the layer adhesion in the (0,0,1) direction significantly affected the tensile strength, since the inter-layer adhesion bonds between adjacent fibres withstood most of the applied load [41,56]. Finally, from the tensile test it is possible to assess that the layer thickness does not highly influence the mechanical properties of the sample in the (1,0,0) direction as it is possible to see from Figure 6.

Figure 7 shows that the layer thickness has an influence on the mechanical properties in the (0,0,1) direction. In particular, samples printed with a layer of 0.6 mm seem to show lower mechanical properties both in terms of σ_max_ and ε_max_, especially for PETG-S and PLA-F. In any case, the mentioned relationship between mechanical properties and layer thickness is not precise enough to fully predict the mechanical behaviour of specimens. This consideration is well supported by the maximum discrepancies (Figure 7c) observed in the case of PETG and PLA samples, where it is possible to observe different behaviour for the different brands considered.

**Figure 8:**
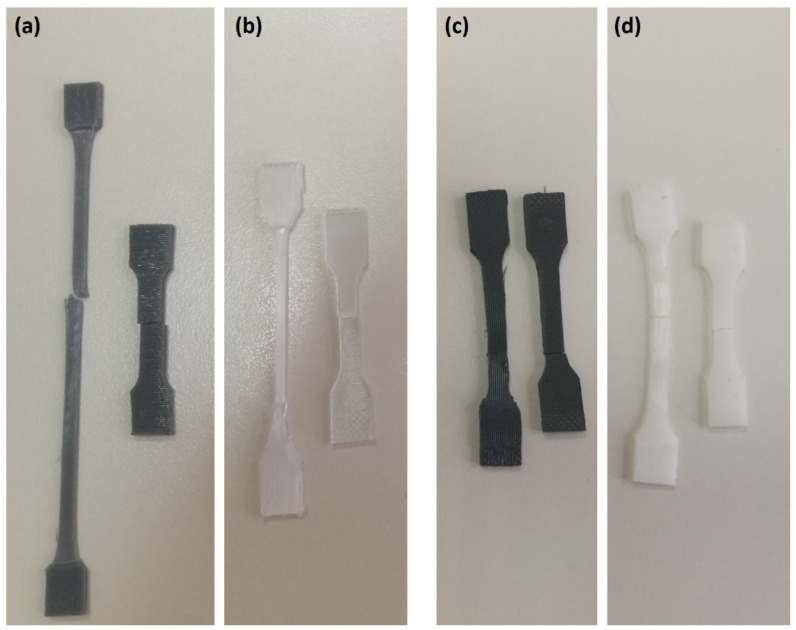
Failure mode for the tested 3D samples: (**a**) PETG-S, (**b**) PETG-F (**c**) PLA-T and (**d**) PLA-F.

The results related to the mechanical behaviour of the samples are supported by the analysis of the failure modes. In Figure 8, it is possible to see that PETG-S showed the best behaviour in terms of elongation at break when tested along fibre direction (1,0,0) (Figure 8a).

The analysis also demonstrates the typical brittle behaviour of PLA, both T and F, with the fracture that occurred without plastic deformations when tested along the fibre direction. When the samples are tested perpendicular to the fibre direction, both PETG and PLA instead demonstrate a typical brittle behaviour (Figure 8b,d), confirming the results of the tensile tests. The low tenacity of the fibre–fibre interface leads to a brittle fracture of the sample even at low deformation.

#### 3.3.2. Charpy Impact Tests 

Charpy tests on printed materials provide an assessment of the amount of energy absorbed by the structure during high-speed impact with a rigid body. This allows for the derivation of the toughness or “fracture resistance” of a 3D printed part as a function of the processing conditions [25,57].

**Figure 9:**
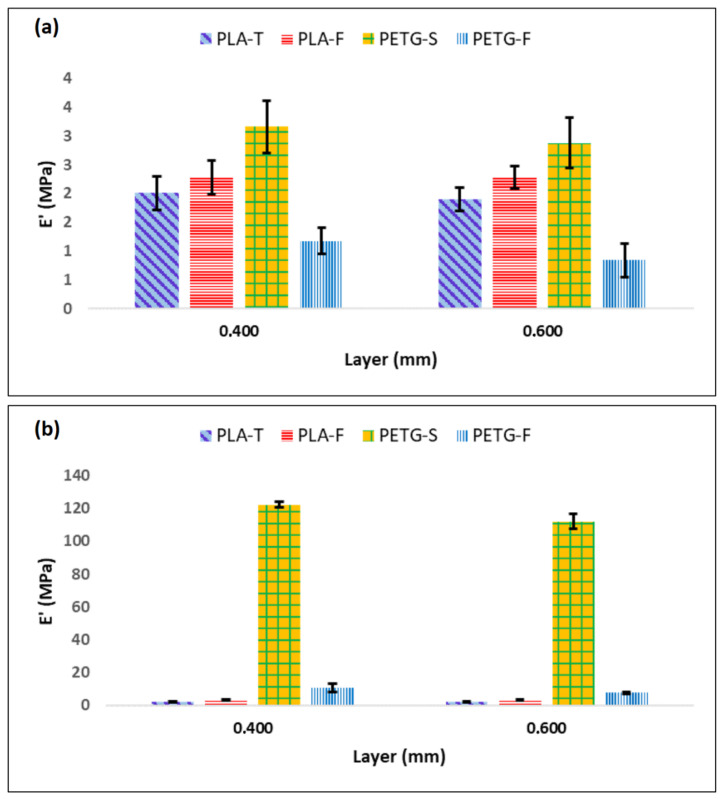
Results of the impact strength in Charpy impact tests on the V-notched specimens of 3D-printed PETG and PLA samples in (**a**) (1,0,0) and (**b**) (0,0,1) fibre orientation.

For each condition analysed, five samples were cut from the hemi-cylinders (Figure 2c) and tested. The test result is typically the average of five tests. From Figure 9 it is possible to notice that both the material and the raster angle have a strong effect on the impact resistance of the samples. The different directions analysed give information on the quality of the inter-layer bonding (direction (1,0,0)) and on the interface bonding between adjacent layers (direction (0,0,1)). The experimental results seem to suggest that the layer thickness has only a small negative influence on the impact resistance of the samples. In particular, a small reduction in impact resistance is observed as the thickness of the layer increases. This behaviour is probably due to the smaller number of inter-layers present in the samples having a greater layer thickness. Fewer interfaces between different layers correspond to a reduced number of interlayer bonds and therefore to a reduction of Ec.

Samples in direction (0,0,1) also show a small reduction in the impact strength with increasing layer thickness. In this case, the impact is perpendicular to the interlayers and therefore the resistance is almost exclusively that of the individual fibres. As the layer thickness increases, the number of layers decreases and therefore the ability to absorb energy during impact is also reduced. The impact resistance of PETG in the direction (0,0,1) was two orders of magnitude higher than that of PLA, going from 122,27 KJ/m^2^ for PETG-S 0.4 mm to 2.28 KJ/m^2^ for PLA-T 0.4 mm. Interestingly, different brands exhibit different mechanical properties. For example, PETG-S demonstrated better impact resistance than PETG-F, confirming what was observed in tensile tests. In any case, it is evident that all the PLA samples show a fragile behaviour in both analysed directions ((0,0,1) and (1,0,0)). This means that for safety reasons, PLA is less suitable than PETG for making back braces. In the event of accidental impact, in fact, the PLA brace could break in a fragile way, exposing sharp parts, while the PETG brace ensures both a higher elongation at break and high absorbed energy.

#### 3.3.3. Dynamic Mechanical Thermal Analysis (DMTA)

**Figure 10:**
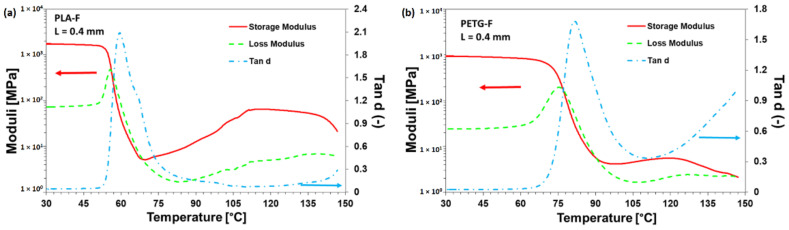
DMTA tests on (**a**) PLA-F and (**b**) PETG-F.

Dynamic temperature sweep tests were performed at the constant frequency of 1 Hz on the 3D-printed samples analysed. Figure 10a,b show the storage modulus (E′), the loss modulus (E″) and the damping factor (tan δ) as a function of temperature for the samples PLA-F and PETG-F. At lower temperature, both samples demonstrated predominantly solid behaviour, with E′ > E″. The storage modulus represents the elastic contribution of the polymer matrix and defines the ability to store energy when deformed, whereas the loss modulus relies on the energy consumed as heat due to internal friction. A maximum value of approximately 1.8 GPa at room temperature was obtained for the storage module of PLA-F. This demonstrates a strong interlocking of the printed filaments, which increases the stiffness of the samples. The stiffness of all samples decreases dramatically as the temperature approaches the glass transition temperature at which the polymer matrix transforms from a solid state to a rubbery state. This behaviour ensures a good adhesion between the layers, as a marked elastic component or incomplete relaxation of the polymer chains after deposition negatively affects the welding between the deposited filaments and thus the mechanical properties of the printed part.

DMTA results for all analysed materials are reported in Table 3. From these results it is deduced that for all samples the glass transition temperature, represented by the peak temperature of the Tan δ curves, shifted to a lower temperature with the increase in layer thickness. This result suggests that higher layer thickness induces lower adhesion between the printed layers and thus lower mechanical stability with temperature. This effect is more evident for the PLA samples whereas it is less important for the high glass transition materials (PETG). It is most likely that PETG filaments are more prone to forming a physical cross-linking network during the deposition phase. This network stabilises the mechanical resistance of the printed parts and stabilises their shape during the cooling phase.

### 3.4. Morphological Analysis

**Figure 11:**
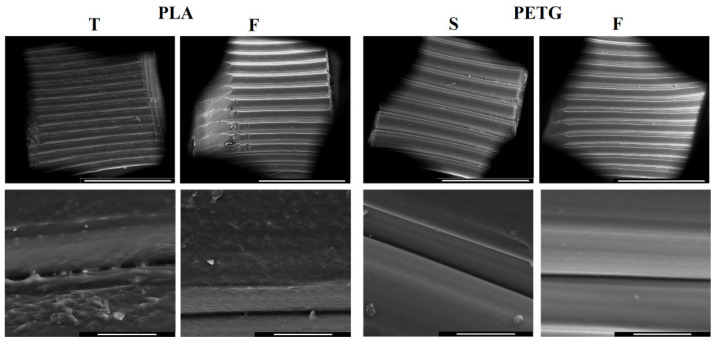
SEM image of the 3D-printed samples showing the morphology for the samples printed with 0.4 mm layer thickness (upper row magnification 100×, scale bar 1 mm; bottom row magnification 1000×, scale bar 100 µm).

The surface morphologies of some selected samples are reported in Figure 11. These micrographs were collected with a SEM at different magnifications (100× and 1000×). In particular, the observation at lower magnification (Figure 10 upper row) clearly demonstrates that the PETG-based samples show a better surface finishing compared to the PLA-based samples.

Furthermore, the PETG-based samples show improved cohesion between layers with a continuous and defect-free interface (bottom row). On the other hand, the PLA-based samples show a surface characterised by numerous macroscopic defects. In this case, the interface between the layers is discontinuous due to the presence of numerous defects. These observations are consistent with the mechanical property results. In fact, the PLA-F samples show better mechanical properties than those of the PLA-T samples. This is consistent with the superior quality of the interface between the layers shown by the PLA-F sample in Figure 11. From these results it is evident that a close correlation exists between the mechanical properties and the morphology of the 3D-printed samples. In particular, the samples obtained using PETG are those with a better surface quality and superior mechanical properties.

### 3.5. Porosity Results

**Figure 12:**
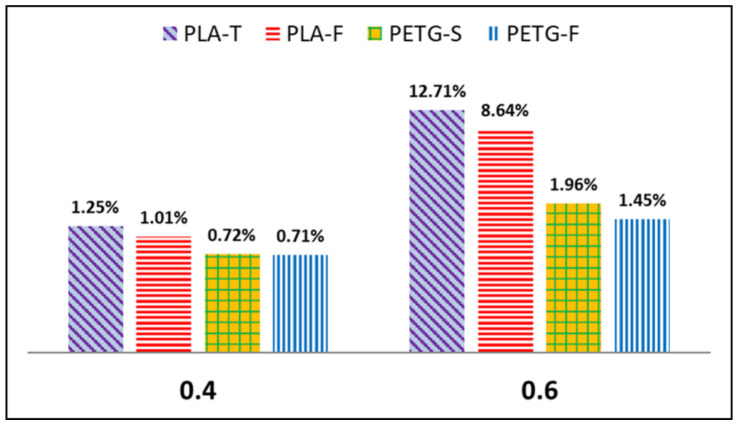
Porosity results of all the samples analysed.

To better evaluate the degree of surface finish and the effective cohesion between the different polymers layers, in Figure 12 we report the results of the samples’ porosity as a function of the layer height. As expected, the porosity increased as the layer height increased. Lower layer heights correspond to a higher deposition pressure and therefore to a more regular adhesion and fewer pores in the structure. The PLA samples show a higher level of porosity than the PETG ones. This result is in agreement with what is evidenced by SEM micrographs and mechanical tests. The PETG-S sample shows a smaller porosity ration. In fact, this sample shows both better surface finish and mechanical properties.

## 4. Discussion

Interest in 3D printing of orthopaedic back braces is growing due to the undeniable benefits it can provide [13]. A high degree of customisation, accurate dimensions and short design time are some of the advantages. 3D printing also allows for the saving of raw materials and reduction of waste. However, some problems must be overcome, including the few materials available, geometric constraints such as the dimensions and arrangement of the piece and the high anisotropy of the mechanical properties of 3D-printed objects. To date, the materials available are rather limited and generally do not present accurate and exhaustive technical data sheets. Often, with the available data, it is difficult to select the material as well as effectively fine-tune the printing parameters. Only through a careful selection of these parameters can effective mechanical and morphological properties be obtained. The need to use amorphous materials or materials with low crystallisation levels considerably limits the mechanical properties that can be obtained. Furthermore, these materials do not offer good stability over time. This can adversely affect the functionality of the finished products. On the other hand, the use of fillers to limit crystallinity and increase mechanical properties can be problematic for biomedical applications. In fact, the use of these materials led to important weight gain and could eventually present problems for body contact. The 3D printing of orthopaedic braces can be considered an effective process only if it allows for the minimisation of post-treatments and manual intervention by specialised operators. Most 3D-printed parts need extensive cleaning operations to remove the backing material and smooth the surface to achieve the required finish. The post-processing may require mechanical sanding, chemical treatments and protective coatings. The amount of post-processing depends on factors including the material used, the printing speed, the height of the layer, the amount of support and the degree of adhesion between the layers. All these treatments can considerably slow down the production of the pieces.

The 3D printing of large pieces (>50 cm) such as orthopaedic braces is still difficult. The majority of 3D printers currently on the market have small print chambers that limit the size of the printable parts. Printing multiple separate parts to be joined together in post-production is currently not a viable approach for these products. In any case, this approach would increase costs and production time and inevitably decrease the quality of the products obtained. Brace printing at the moment requires quite high printing times (longer than the duration of a work shift). This means that, if the printer is not equipped with a thermo-regulated printing chamber, the piece is made in variable conditions of temperature and humidity. The cooling conditions of the deposited material are also very variable between the different areas of the brace. This is because commercial printers are not equipped with cooling fans that can be directed according to the printing position. Finally, there are still no shared technical specifications on the development of 3D printers and control software. This does not guarantee the right level of quality and accuracy of the printed pieces. Despite all the above, 3D printing of orthopaedic braces remains an important opportunity to increase the quality and therapeutic functionality of these devices. Most of the highlighted limitations can be solved with the normal development of technology and materials. In a few years, a back brace obtained via 3D printing can become competitive with those obtained by thermoforming.

## 5. Conclusions

This work presents a complete characterisation of two types of polymeric materials (PLA and PETG) potentially usable for making orthopaedic back braces. The 3D printing of back braces has great potential, however additional knowledge is necessary for the correct design and manufacture of these devices. This is particularly true when it comes to the choice of material and the optimal process conditions for making the back braces. The right material must allow for the creation of a light back brace while ensuring adequate resistance over time, optimal positioning, excellent biocompatibility and a good level of thermo-hygrometric comfort. The results obtained in this work show that layer thickness and raster orientation must be considered to optimise the printing process of back braces. In fact, it has been found that the 3D-printed samples have quite different mechanical behaviours if tested along the fibre direction (1,0,0) or perpendicular to fibre direction (0,0,1). This is important information, helpful for deciding the printing parameters used for the realisation of a scoliosis back brace. Beyond mechanical properties, the morphology also affects both the quality and the performance of the printed parts, which is why we conducted an accurate morphological investigation to show that PETG not only has superior mechanical properties but also better finishing when compared with PLA. These results have a direct influence on the printing parameters and the material choice for the realisation of a scoliosis back-brace. However, the conditions analysed give a rather limited view of the real conditions. Further work must be considered to cover all the work conditions that the brace will undergo.

## Figures and Tables

**Table 1:** Properties of the considered polymers using data from the database CES EduPack (Granta Design, UK) and filament datasheets.

	PLA		PETG	
***Tradename***	***Filoalfa^®^***	***TreeD Filaments^®^***	***Filoalfa^®^***	***Sunlu^®^***
***Short-name***	PLA-F	PLA-T	PETG-F	PETG-S
***Tensile strength (ASTM D638)***	53 MPa	53 MPa	50 MPa	44 MPa
***Density (ASTM D792)***	1.24 g/cm^3^	1.24 g/cm^3^	1.27 g/cm^3^	
***Tensile modulus (ASTM D882)***	3600 MPa	3050 MPa		2110 MPa
***Flexure modulus (ASTM D790)***	3800 MPa		2110 MPa	
***Flexure strength (ASTM D790)***			73 MPa	
***Elongation at break (ASTM D882)***	6%			18%
***IZOD impact strength (ASTM D256)***			100 J/m	
***Notched IZOD impact***		16 J/m		
***Heat deflection temperature*** ***(ASTM D648)***	55 °C	55 °C	70 °C	73 °C
***Melt flow rate***				6–11 g/10 min (220 °C, 2.16 kg)
***Price*** **(per kg)**	15–25€	15–25€	20–40€	20–40€

**Table 2:** Properties of the considered polymers.

SAMPLE		T_g_	T_m_	ΔH_m_
		(°C)	(°C)	(J/g)
**PETG-S**	Pre	70.8	-	-
	Post	71.6	-	-
**PETG-F**	Pre	73.9	-	-
	Post	77.3	-	-
**PLA-F**	Pre	62.4	152.7	14.9
	Post	58.5	150.6	23.9
**PLA-T**	Pre	60.9	149.0	12.4
	Post	59.7	147.3	17.7

**Table 3:** DMTA results of the considered polymer samples.

Sample		PLA-F		PLA-T		PETG-F		PETG-S	
**Layer Thickness**		**0.4**	**0.6**	**0.4**	**0.6**	**0.4**	**0.6**	**0.4**	**0.6**
**Tg (°C)**		61.1	59.6	59.0	56.1	84.5	81.7	79.3	79.1
**E′** **(MPa)**	**30 °C**	1733	1394	1269	776	937	837	951	891
	**55 °C**	982	1050	53	227	868	772	879	812
	**80 °C**	7	3	6	7	52	99	14	23
**E″** **(MPa)**	**30 °C**	73	51	40	53	24	22	28	23
	**55 °C**	399	168	111	150	26	23	30	22
	**80 °C**	2	1	1	2	82	117	25	36

## Data Availability

Data are available on request by sending an email to the corresponding author.
